# How the U.S. presidential election impacts global health: governance, funding, and beyond

**DOI:** 10.1186/s41256-024-00391-w

**Published:** 2024-11-20

**Authors:** Yuming Liu, Brian J. Hall, Minghui Ren

**Affiliations:** 1 School of Public Health, Peking University, Xueyuan Road No. 38, Haidian District, Beijing, 100191 China; 2 Center for Global Health Equity, New York University Shanghai, Shanghai, China; 3 Department of Global and Environmental Health, New York University School of Global Public Health, New York, USA; 4 Health, Behavior, and Society, Johns Hopkins University Bloomberg School of Public Health, Baltimore, USA; 5 Institute for Global Health, Peking University, Beijing, China; 6 China Center for Health Development Studies, Peking University, Beijing, China

**Keywords:** Global health, Global health governance, Global health diplomacy, Health equity, US election

## Abstract

The United States plays a crucial role in shaping global health through its policy decisions and engagements. Historically, bipartisan support underpinned U.S. involvement in multilateral and bilateral global health initiatives in advancing its national health, security interests, and foreign policy. However, recent decades have witnessed increased politicization and polarization of global health and fluctuating stances between Republican and Democratic administrations. This commentary speculates the potential implications of the 2024 presidential election on global health, focusing on how ideological differences between parties and previous administrative actions might affect U.S.’s strategies in addressing key global health issues, including governance, funding allocation, sexual and reproductive health policies, responses to humanitarian crises, and efforts to combat climate change. The election may be a critical juncture that could determine whether the U.S. global health strategies will continue to reflect the globalist and liberal policies typically associated with recent Democratic administrations or shift back to the isolationist tendencies observed during Trump’s presidency. The outcome will significantly determine the direction of U.S. global health policy and its broader implications for global health equity and security. The conclusions emphasize the necessity of maintaining strong international cooperation and commitment to health as a global public good.

## Background

In today’s environment, health challenges require a global approach for sustainable solutions. As a leading actor in global health, the United States significantly influences international programs and outcomes through policy decisions, financial contributions, and collaborations. The upcoming U.S. presidential election poses potential shifts that could drastically affect the global health dynamics and impact populations worldwide. Understanding how U.S. policy can affect governance structure and international collaboration is necessary for anticipating and navigating future challenges in global health.

This perspective discusses the possible impacts of the presidential election on global health. Given that the detailed global health strategies of the Trump and Harris tickets remain unknown, this analysis will be speculative, drawing on persistent ideological differences between the Republican and Democratic parties and policies introduced during Biden’s administration and those from Trump’s first term. We aim to forecast potential shifts in U.S. strategies addressing key global health issues.

## U.S. involvement in global health: multilateral and bilateral engagements

In the decades following World War II, U.S. engagement in global health enjoyed bipartisan support, and the country played a pivotal role in establishing key health organizations under the United Nations (UN) umbrella, notably facilitating the creation of the World Health Organization (WHO) [1]. As U.S. priorities in global health evolved, the nation also championed public–private partnerships, such as Gavi, the Vaccine Alliance, and the Global Fund [2].

Bilateral engagement is also a key aspect of U.S. foreign policy in health, utilizing platforms such as the United States Agency for International Development, the Fogarty International Center at the National Institutes of Health, and the President’s Emergency Plan for AIDS Relief (PEPFAR). These initiatives protect U.S. national interests and support ethical standards [2]. Additionally, they support broader U.S. foreign policy objectives, including poverty reduction, economic development, peace promotion, and improving the country’s global stature.

The politicization of global health intensified with President Reagan’s election in 1980, marked by his administration’s withdrawal from the United Nations Educational, Scientific and Cultural Organization and reluctance to support WHO-driven multilateral initiatives [3]. This period saw delayed U.S. funding to the WHO during the emerging AIDS pandemic, exacerbated by Congressional delays, resulting in WHO experiencing its most severe budget crisis, adversely affecting global health equity [4]. The era also introduced the “Mexico City Policy,” or the “Global Gag Rule (GGR),” prohibiting U.S. assistance to foreign non-governmental organizations involved in abortion-related services [5]. U.S. global health policy has since fluctuated with political leadership: Republican administrations reinstating the GGR and reducing global health commitments, while Democratic counterparts have rescinded the GGR and increased global contributions [1]. Despite these restrictions, Republican-led initiatives like PEPFAR and the President’s Malaria Initiative (PMI), both launched under the George W. Bush administration, illustrate that partisan policies can simultaneously restrict and advance U.S. global health efforts, reflecting a complex interplay of ideological beliefs and health contributions.

## Global health approaches under the Trump and Biden administrations

The ideological shift in U.S. policy toward global health governance have been stark between the Trump and Biden administrations. During his previous term, the Trump administration shifted toward bilateral engagement, fueled by an “America First” ideology that viewed international organizations as limitations on U.S. sovereignty [1, 6]. Such ideologies, influenced more by international relations over global health concerns, resulted in reduced international involvement, withdrawal from the Paris Agreement, expanded prohibitions under the GGR, and decreased funding for global health initiatives. This approach also resulted in a regression in global health efforts and eroded much of the trust and leadership the U.S. had previously built, turning its historical contribution into a “negative asset” on the global stage. Trump’s actions during COVID-19, such as withdrawing from the WHO and opting out of COVID-19 Vaccines Global Access, damaged global solidarity and health security [6].

In contrast, upon entering office, the Biden administration reversed many of Trump’s policies, rejoining the WHO, repealing the GGR, reentering the Paris Agreement, and renewing commitments to global partnerships. This marked an effort to reassert U.S. leadership in global health by reconnecting with the WHO and advocating for broader international health regulatory oversight [2].

## Electoral impact on U.S. global health policy and international relations

The 2024 election again shapes the country’s engagement in global health, particularly its relationship with the WHO and other UN agency-affiliated health programs. The U.S. has traditionally held leadership positions within these organizations, a status that was diminished during Trump’s presidency [7]. The election could further complicate the future of the International Health Regulations and the ongoing negotiations for the Pandemic Agreement. A potential return of Trump could see a refocus on the origins of COVID-19 outbreak and escalated tensions with China, which may endanger mutual trust, global solidarity and hinder collaboration needed to address emerging challenges, including the threat of a new global pandemic of disease X, climate change and health, and antimicrobial resistance. Regardless of electoral outcomes, maintaining U.S.-China collaboration is essential for global health security. This includes ongoing partnerships like the annual U.S. Center for Disease Control and Prevention (CDC) and China CDC meetings, and joint responses to crises such as the 2013 Ebola outbreak.

The electoral outcome will shape global health funding, particularly affecting low- and middle-income countries (LMICs). The U.S. significantly impacts global health, funding programs like PEPFAR and PMI, alongside supporting the Global Fund, WHO, and the Joint United Nations Programme on HIV/AIDS to boost global health security and preparedness [8]. However, these commitments are sensitive to political change; previously under the Trump administration, significant funding cutbacks introduced uncertainty, unsustainability, and disrupted ongoing health initiatives [1, 6, 8]. A potential withdrawal during a second Trump term would exacerbate these issues, potentially leading to another financial crisis for the WHO. The U.S. government provided 22% of the assessed contributions and about 500 million USD in voluntary contribution to the WHO base segment for 2022–2023 [9]. Such a withdrawal would force other member states and partners to cover the deficits, posing greater risks to public health security globally. Therefore, strategic financial investments not only strengthen international relationships and build trust but are also important for sustaining long-term health outcomes and the ability to influence global health policies and practices.

The GGR exemplifies conservative views on sexual and reproductive health. The Trump administration expanded it to include all family planning services, profoundly affecting global health initiatives across domains including HIV/AIDS, malaria, Zika, maternal and child health, and nutrition [1, 2]. The expansion fractured links within healthcare systems and fragmented care integration crucial in LMICs—evident in deteriorated healthcare access, coordination, and communication [5, 10]. Following historical patterns, the Biden administration reversed the GGR [1]. Such fluctuations pose challenges to sustaining long-term global health programs and respect of human rights.

Ideological differences between U.S. political parties are reflected in their approaches to global humanitarian crises. Human rights have been a cornerstone of U.S. foreign policy, but the Trump administration reduced these commitments by withdrawing from the UN Human Rights Council [1, 6]. The Biden administration reinstated U.S. membership. Despite this re-engagement, both administrations faced criticism for insufficient accountability measures regarding international humanitarian law violations and placing geopolitical agendas above adherence to international law. The decisions of future administration will critically influence the U.S.’s role in shaping global values.

The impact of climate change on global health is increasingly influenced by U.S. environmental policies, which remain divided along partisan lines. Echoing previous Republican administrations, the Trump administration downplayed the threat of climate change and questioned human impacts on environmental challenges. Emphasizing free-market ideologies, it prioritized deregulation, rolled back numerous environmental regulations, reduced renewable energy initiatives, and supported the fossil fuel industry [7]. This approach was epitomized by the U.S.’s withdrawal from the Paris Agreement, rationalized by concerns over “energy dominance.” President Trump has signaled intentions to further increase deregulation should he return to office. In contrast, the Biden administration has re-embraced the goals of the Paris Agreement and pledged to reengage the U.S. with broader global efforts addressing climate change, biodiversity, and environmental sustainability [8]. The stance of the future U.S. administration will influence global climate dynamics, determining whether its approach is more cooperative or competitive on the international stage.

## Conclusions

The 2024 U.S. Presidential election is approaching and will shape the country’s engagement in global health. Although details of each candidate’s global health strategies remain undisclosed, ideological differences between the Trump and Harris tickets will likely mirror previous partisan approaches, yet their implementation will require navigating the complex dynamics of interbranch cooperation involving the legislative, executive, and judiciary branches. This interaction influences international representation and resource allocation in global health. Moreover, partisan divisions and ideological shifts have eroded essential academic partnerships, emphasizing the need for guardrails that separate areas of competition from those where cooperation, like global health, should be strengthened. Regardless of the electoral results, profound implications for global health equity are anticipated, emphasizing the necessity for the U.S. to reassert its commitment to treating health as a global public good through concerted efforts.

## Data Availability

Not applicable.

## Abbreviations

UN: United Nations
WHO: World Health Organization
PEPFAR: U.S. president’s emergency plan for AIDS relief
PMI: President’s malaria initiative
AIDS: Acquired immunodeficiency syndrome
GGR: Global gag rule
CDC: Center for disease control and prevention
LMIC: Low- and middle-income country
HIV/AIDS: Human immunodeficiency virus infection and acquired immunodeficiency syndrome

## References

[1] Meier BM, Palmquist A, Dockery M, Saggi N, Ekeigwe K, Latorre I, et al. The 2024 U.S. elections: global health policy at a crossroads. J Law Med Ethics. 2024;70:1069. 10.1017/jme.2024.106 39435917

[2] Noon MJ, She X, Meier BM. The US elections as a determinant of global health. Lancet Reg Health Am. 2024;39:100884. 39309538 10.1016/j.lana.2024.100884 PMC11416580

[3] Massing M. Unesco under fire. Atlantic. 1984;254:88–97.

[4] Thomas KK. The other time a U.S. president withheld WHO funds: johns hopkins Bloomberg school of public health; 2020. https://publichealth.jhu.edu/2020/the-other-time-a-us-president-withheld-who-funds.

[5] Barot S. When antiabortion ideology turns into foreign policy: How the global gag rule erodes health, ethics and democracy: Guttmacher; 2017. https://www.guttmacher.org/gpr/2017/06/when-antiabortion-ideology-turns-foreign-policy-how-global-gag-rule-erodes-health-ethics.

[6] Stivachtis YA. Trump administration’s approach to global health governance. In: Akande A, editor. The perils of populism: the end of the american century? Cham: Springer Nature, Switzerland; 2023. p. 379–402.

[7] Ratevosian J, Yamey G. The presidential election will shape the future of human health time magazine; 2024. https://time.com/7027503/harris-trump-election-global-health/.

[8] Meier BM, Noon MJ, Barry M, Skusker P, Palmquist A, Saggi N, et al. Election 2024 fact sheets: an existential crossroads for global health. Consortium of Universities for Global Health; 2024.

[9] World Health Organization. All for health, health for all: investment case 2025–2028. Geneva: World Health Organization; 2024.

[10] Mavodza C, Goldman R, Cooper B. The impacts of the global gag rule on global health: a scoping review. Global Health Res Policy. 2019;4(1):26. 10.1186/s41256-019-0113-3 PMC6714436 31485484

